# An antibody panel for highly specific detection and differentiation of Zika virus

**DOI:** 10.1038/s41598-020-68635-6

**Published:** 2020-07-17

**Authors:** Md Alamgir Kabir, Ruben Soto-Acosta, Sandhya Sharma, Shelton S. Bradrick, Mariano A. Garcia-Blanco, Massimo Caputi, Waseem Asghar

**Affiliations:** 1 Department of Computer & Electrical Engineering and Computer Science, Florida Atlantic University, Boca Raton, FL 33431 USA; 2 Asghar-Lab, Micro and Nanotechnology in Medicine, College of Engineering and Computer Science, Boca Raton, FL 33431 USA; 3 Department of Biochemistry & Molecular Biology, University of Texas Medical Branch, Galveston, TX USA; 4 Duke-NUS Medical School, Singapore, Singapore; 5 Department of Biomedical Science, Charles E. Schmidt College of Medicine, Florida Atlantic University, Boca Raton, FL 33431 USA; 6 Department of Biological Sciences (Courtesy Appointment), Florida Atlantic University, Boca Raton, FL 33431 USA; 7 Present Address: Center for Drug Design, University of Minnesota, 312 Church Street, Minneapolis, MN 55455 USA; 8 Present Address: MRIGlobal, Kansas City, MO USA

**Keywords:** Immunoblotting, Viral infection

## Abstract

Zika virus (ZIKV) is an emerging flavivirus transmitted to humans by Aedes mosquitos. ZIKV can be transmitted from mother to fetus during pregnancy and can cause microcephaly and other birth defects. Effective vaccines for Zika are yet to be approved. Detection of the ZIKV is based on serological testing that often shows cross-reactivity with the Dengue virus (DENV) and other flaviviruses. We aimed to assemble a highly specific anti-Zika antibody panel to be utilized in the development of a highly specific and cost-effective ZIKV rapid quantification assay for viral load monitoring at point-of-care settings. To this end, we tested the affinity and specificity of twenty one commercially available monoclonal and polyclonal antibodies against ZIKV and DENV envelope proteins utilizing nine ZIKV and twelve DENV strains. We finalized and tested a panel of five antibodies for the specific detection and differentiation of ZIKV and DENV infected samples.

## Introduction

Isolated in 1947 from a rhesus monkey^1^ the Zika virus (ZIKV) is a member of the virus family Flaviviridae (genus Flavivirus). ZIKV is transmitted by various species of *Aedes* mosquitoes^2,3^ and was not considered critical to global health until the last decade, after a series of outbreaks on several Pacific islands^4–7^. In the United States, the first outbreak of Zika was reported in 2016 with a total of 5,168 symptomatic cases in the continental US and 35,395 cases in Puerto Rico^8,9^. In 2015 and 2016, total 1,673,272 cases were reported in Brazil^10^. To date, Zika virus has circulated to all continents with the exception of Europe and Antarctica^11^. Without the availability of an effective vaccine the prevention of disease transmission is dependent on the early diagnosis of the virus to determine the site and size of an outbreak and the effectiveness of vector control measures^12^.

ZIKV is an enveloped virus with a single-stranded, positive sense RNA genome coding for three structural proteins (C, prM/M, and E) and seven non-structural proteins (NS1, NS2A, NS2B, NS3, NS4A, NS4B, and NS5)^13^. Dengue viruses (DENV1-4), of the genus Flavivirus, are structurally and genetically related to ZIKV^14,15^ and are also transmitted by *Aedes* spp. mosquitoes^14^. The envelope (E) protein of both ZIKV and DENV, is highly immunogenic and is expressed on the surface of the virus to mediate the binding and the membrane fusion of the target cell^16,17^.

The viral envelope protein consists of three main domains (ED I, II, III). Neutralizing antibodies (primarily IgG class) against the ED I and ED II domains are more prone to show cross-reactivity between DENV and ZIKV due to a higher homology (ED I (35%) and ED II (51%)) compared to the ED III domain (29%)^18^. As a result of the considerable structural and genetic similarities between ZIKV and DENV, neutralizing antibodies often show cross-reactivity in serological assays. Immunological cross-reactivity between ZIKV and DENV have already been reported during the Yap State (Micronesia) outbreak^19^. Furthermore, in the Americas and Africa, DENV and yellow fever virus, also a member of the Flaviviridae family, can be found in the same geographical areas, and DENV present symptoms similar to ZIKV, hence a precise differential diagnosis among these viruses is critical to implement the proper monitoring and prevention strategies^20–23^.

Currently, identification of ZIKV infection is accomplished by i) testing the serum to detect viral nucleic acid using RT-PCR, ii) testing the serum for the presence of the non-structural 1 (NS1) protein antigen or iii) serological assays to determine the presence of virus-specific immunoglobulin IgG and IgM antibodies using enzyme-linked immunosorbent assay (ELISA)^3,24^. Unfortunately, ZIKV IgM-ELISA displays high specificity, but poor sensitivity, while the ZIKV IgG-ELISA are characterized by low specificity and cross-reactivity in patients previously exposed to dengue infections^25^. Other assays, such as the plaque reduction neutralization test (PRNT), can be performed to measure virus-specific neutralizing antibodies but show high accuracy only after day 7 of the disease onset^2,26–28^, are labor-intensive, expensive and time-consuming. Similarly, RT-PCR assays, although highly specific^29–31^, are expensive and require multiple labor-intensive sample preparation steps. Considering all the factors and limitations of the Zika detection methods currently utilized, there is an unmet need to develop a rapid, inexpensive, minimally labor-intensive, and highly specific detection assay for ZIKV that can be utilized in point of care settings without the access to specialized equipment and facilities.

In the USA, ZIKV diagnostic assays for either detection of antibodies or nucleic acid from ZIKV were not available before 2016. Since 2016 the FDA has issued an Emergency Use Authorization (EUA) for fourteen molecular-based assays for the detection of genetic material in samples of bodily fluids, such as serum and urine, along with five serological-based assays for the detection of antibodies against ZIKV in the blood^32^. Among the assays one (CDC Zika MAC-ELISA) utilizes noninfectious ZIKV-like particles, another one (InBios) uses recombinant ZIKV E glycoprotein whereas the rest utilize recombinant ZIKV NS1 antigen^33^. Although individual serologic assays have a prolonged window of detection, they also have disadvantages. The CDC and the InBios assays show lower specificity^34^ due to the similarities in the antigenic structure of the E protein between DENV and ZIKV while the majority of the other assays shows lower sensitivity^34–36^. The current ZIKV serological assays only comprise IgM class antibodies and to date, there is no FDA EUA approved IgG based ELISA assay due to the higher potentiality of cross-reactivity. CDC guideline suggests additional PRNT testing if a sample results are positive with any of the above-mentioned assays^37^. Therefore, the development of tools that allow ZIKV E protein detection in various experimental conditions is of utmost importance. The selection of highly specific and non-cross-reactive antibodies is the first step for the development of an effective detection platform for the ZIKV.

In this study, we have evaluated the sera cross-reactivity of twenty one monoclonal and polyclonal antibodies against the ZIKV and DENV E proteins to assemble a highly specific panel of antibodies for the specific detection and differentiation of ZIKV from DENV. The antibodies were tested against nine ZIKV and twelve DENV strains. Next, the panel of selected antibodies were tested with deidentified ZIKV and DENV viral culture lysates and their lower limit of detection was determined by western blot.

## Materials and methods

### Antibodies and viruses

We utilized twenty one commercially available antibodies purified from either mouse hybridoma cell line or rabbit against the ZIKV or DENV E protein (Table S1). HRP-conjugated anti-rabbit IgG and anti-mouse IgG antibodies were used as secondary antibodies (GE Healthcare Life Science). Twelve different strains of DENV and nine different strains of ZIKV were obtained from the ATCC and BEI resources repositories (Table S2). Four virus samples (one DENV and three ZIKV) were quantified by foci-forming assay^38^ and plaque assay^39^ and utilized for the antibody panel specificity and affinity testing.

### Cell culture

Vero cells were grown in Dulbecco's Modified Eagle Medium with 10% Fetal Bovine Serum and 1% Gentamicin at 37° C in 5% CO2.

### SDS-PAGE and western blot

ZIKV/DENV samples were mixed with 2 × Laemmli Sample Buffer containing 10% β-mercaptoethanol and heated at 95 °C for 5 min. Samples containing either ZIKV or DENV were separated utilizing a 10% SDS–polyacrylamide, electroblotted onto a nitrocellulose membrane (Thermofisher). The membrane was blocked at room temperature for 30 min in 5% milk–TBST (50 mM Tris, 150 mM NaCl and 0.2% Tween-20) and then, probed with the primary antibodies (60 min) listed in Table 1, the HRP conjugated secondary antibody (1:5,000 dilution in 2.5% milk–TBST, for 60 min) and stained using the Supersignal West Femto Maximum Sensitivity substrate (Thermofisher). Luminescence was quantified utilizing an Odyssey classic imaging system (LICOR Biosciences, Bad Homburg, Germany). All assays were run as independent duplicates.

**Table 1:** Assessment of specificity and cross-reactivity of the tested antibodies against nine ZIKV isolates.

Catalog No	NR-50234	VR-1838	VR -1843	NR-50183	NR-50245	NR-50280	NR-50355	NR-50066	NR-50551
NR-2556 (D)	–	–	–	–	–	–	–	–	–
10-1706 (D)	–	–	–	–	–	–	–	–	–
NR-4757 (D)	–	–	–	–	–	–	–	–	–
10-1435 (D)	–	–	–	–	–	–	–	–	–
GTX629116 (D)	–	–	–	–	–	–	–	–	–
GTX127277 (D)^@^	–	–	–	–	–	–	–	–	–
GTX629117 (D)	–	–	–	–	–	–	–	–	–
ab80914 (D)	–	–	–	–	–	–	–	–	–
ab214335 (F)	–	–	–	–	–	–	–	–	–
NR-50327 (F)	–	–	–	–	–	–	–	–	–
NR-50414 (Z)	–	–	–	–	–	–	–	–	–
GTX133314 (Z)^@^	0.544 (+)	2.79 (++)	0.952 (+)	1.50 (+)	0.747 (+)	0.429 (+)	3.25 (++)	0.921 (+)	4.16 (++)
GTX634155 (Z)	0.705 (+)	2.72 (++)	0.398 (+)	0.288 (+)	0.271 (+)	0.155 (+)	0.894 (+)	0.12 (+)	0.509 (+)
BF-1176-56 (Z)^@^	1.72 (+)	5.31 (++)	1.88 (+)	1.58 (+)	1.94 (+)	1.34 (+)	3.13 (++)	1.14 (+)	4.32 (++)
MBS5304716 (Z)	0.349 (+)	0.47 (+)	0.129 (+)	0.385 (+)	0.151 (+)	0.0787 (+)	0.591 (+)	0.0663 (+)	0.849 (+)
GTX133325 (Z)	1.35 (+)	4.28 (++)	2.75 (++)	3.57 (++)	3.48 (++)	2.85 (++)	5.37 (++)	0.815 (+)	6.30 (++)
GTX133326 (Z) *^@^	12.9 (+++)	15.9 (+++)	9.47 (++)	10.7 (+++)	12.7 (+++)	10.6 (+++)	14.1 (+++)	5.33 (++)	19.3 (+++)
GTX634157 (Z)^@^	0.168 (+)	0.626 (+)	0.088 (+)	0.0486 (+)	0.0594 (+)	0.0368 (+)	0.316 (+)	0.101 (+)	0.344 (+)
367950 (Z)	**	**	**	**	**	**	**	**	**
10-2715 (Z)	**	**	**	**	**	**	**	**	**
10-2714 (Z)	**	**	**	**	**	**	**	**	**

The table summarizes the results obtained by testing the ability of 21 antibodies to detect the ZIKV E protein in SDS-PAGE/Western Blot assays. (D) Anti-Dengue virus E protein, (Z) Anti-Zika virus E protein, (F) Anti pan-Flavivirus E protein, the number represents the pixel intensity of the signaling band, (–) no virus detected, (+) low detection with pixel intensity < 2, (++) good detection with pixel intensity > 2 and < 10, (+++) excellent detection with pixel intensity > 10. *Lower MW protein detected in Both Dengue and Zika samples. **A higher MW protein detected in both Dengue and Zika samples, ^@^Indicates Antibody selected for the ZIKV/DENV panel.

### RNA extraction, cDNA synthesis, and real-time qPCR

RNA was extracted using Trizol Reagent (Invitrogen) according to the manufacturer’s protocol. 11 µl of RNA (out of total 50 µl of RNA) was used for cDNA synthesis with SuperScript Reverse Transcriptase system (Invitrogen, California, USA). 2 µl of (1:3 diluted) synthesized cDNA was used for RT-qPCR analysis using Green-2-Go qPCR mastermix (Bio Basic, New York, USA.) with a pair of primers specific for NR–50355 and NR-50245 genomic sequences (5′-GCAAACTGTCGTGGTTCTAG-3′, 5′-CTTTGCACCATCCATCTCAG-3′). Synthesized DNA from a conserved 429 nt region of the ZIKV genome was used as standard. PCR amplifications were performed on an AriaMx Real-time PCR System (Agilent, California, USA) for thermal cycling and SYBR detection with three technical replicates for each sample. The quantification of the ZIKV samples was determined by comparing the cycle threshold (Cq) value based on the standard curve generated by the known DNA samples amount.

## Results

### Validation of the specificity of anti-ZIKV and anti-DENV E protein antibodies

We obtained 19 antibodies raised against either the ZIKV or DENV Envelope (E) protein and two pan-Flavivirus (anti-E protein) antibody with specificity for DENV-1, 2, 3, 4, Japanese encephalitis virus, West Nile virus, yellow fever virus and ZIKV (Table S1). We obtained tissue culture supernatants for twelve different strains of DENV (subtypes 1–4) and nine different strains of ZIKV from the ATCC and BEI Resources repositories (Table S2). E protein amino acid sequences were aligned for both ZIKV/DENV strains using Clustal Omega 2.1 to determine their structural differences/identities with each other (Supplementary Figs. S1 and S2). Moreover, phylogenetic analysis was performed to show evolutionary relationships for all ZIKV/DENV E proteins (Supplementary Fig. S3). The protein quantity of each viral lysates was quantified using standard BCA protein assay reagent (Pierce, Rockford, IL, USA) in 96 well plates following the manufacturer guidelines (Supplementary Fig. S4). Five ZIKV viral isolates exhibited the same E sequence which are from central/south America region, nevertheless their viral preparations and the titer are different. SDS PAGE/Western blot assays were carried out for all the possible antibody/viral strain combinations to determine the specificity and cross-reactivity of all the antibodies tested (Supplementary Figs. S5 and S6). A western blot approach was chosen given the higher sensitivity and specificity compared to standard ELISA assays and other quantitative/qualitative immunohistochemical techniques. Although this technique might display lower sensitivity if the antibody recognizes with high affinity a higher-order structural conformation of the epitope. Tables 1 and 2 summarize the results of the antibodies tested with the ZIKV and DENV strains respectively.

**Table 2:** Assessment of specificity and cross-reactivity of the tested antibodies against nine DENV isolates.

Catalog No	VR-1586	NR-3787	NR-3782	NR-82	VR-1584	NR-12217	NR-49750	NR-84	NR-80	NR-3798	NR-86	NR-49757
NR-2556 (D)	–	–	–	–	–	–	–	–	–	–	–	–
10-1706 (D)	–	–	–	–	–	–	–	–	–	–	–	–
NR-4757 (D)	–	–	–	–	–	–	–	–	–	–	–	–
10–1,435 (D)	–	–	–	–	–	–	–	–	–	–	–	–
GTX629116 (D)	–	–	–	–	–	–	–	–	–	–	–	–
GTX127277 (D) ^@^	–	–	–	–	0.283 (+)	0.192 (+)	0.383 (+)	–	–	–	–	–
GTX629117 (D)	–	–	–	–	0.182 (+)	0.016 (+)	0.031 (+)	–	0.012 (+)	0.013 (+)	–	–
ab80914 (D)	0.164 (+)	0.197 (+)	–	0.276 (+)	–	0.048 (+)	–	–	–	–	–	–
ab214335 (F)	–	–	–	–	–	–	–	–	–	–	–	–
NR-50327 (F)	–	–	–	–	–	–	–	–	–	–	–	–
NR-50414 (Z)	–	–	–	–	–	–	–	–	–	–	–	–
GTX133314 (Z) ^@^	–	–	–	–	–	–	–	–	–	–	–	–
GTX634155 (Z)	–	–	–	–	–	–	–	–	–	–	–	–
BF-1176–56 (Z) ^@^	–	–	–	–	–	–	–	–	–	–	–	–
MBS 5,304,716 (Z)	–	–	–	–	–	–	–	–	–	–	–	–
GTX133325 (Z)	–	–	–	–	–	–	–	–	–	–	–	–
GTX133326 (Z)*^@^	2.20 (++)	2.03 (++)	0.200 (+)	3.49 (++)	0.07 (+)	0.239 (+)	0.234 (+)	–	3.38 (++)	–	–	–
GTX634157 (Z) @	–	–	–	–	–	–	–	–	–	–	–	–
367950 (Z)	**	**	**	**	**	**	**	**	**	**	**	**
10-2715 (Z)	**	**	**	**	**	**	**	**	**	**	**	**
10-27154 (Z)	**	**	**	**	**	**	**	**	**	**	**	**

The table summarizes the results obtained by testing the specificity of 21 antibodies for the DENV E protein in SDS-PAGE/Western Blot assays. (D) Anti-Dengue virus E protein, (Z) Anti-Zika virus E protein, (F) Anti pan-Flavivirus E protein, the number represents the pixel intensity of the signaling band, (–) no virus detected, (+) low detection with pixel intensity < 2, (++) good detection with pixel intensity > 2 and < 10, (+++) excellent detection with pixel intensity > 10. *Lower MW protein detected in Both Dengue and Zika samples. **A higher MW protein detected in both Dengue and Zika samples, ^@^indicates Antibody selected for the ZIKV/DENV panel.

13 out of the 21 the antibodies tested showed reactivity with the E protein from either ZIKV, DENV or both (Figs. S5, S6 and Tables 1, 2). Ten out of the eleven anti-Zika antibodies demonstrate good to excellent reactivity with the ZIKV E protein (Fig. S5, Table 2); however, four of these antibodies displayed marked cross-reactivity with multiple DENV strains. One anti-ZIKV antibodies did not recognize either DENV or ZIKV E proteins. Three of the anti-Zika antibodies that specifically recognized the ZIKV E protein also recognized a higher molecular weight (MW) protein, possibly of cellular origin, but did not cross-react with the DENV E protein. Only three of the eight anti DENV E protein antibodies tested recognized one or more of the DENV strains, but none exhibited cross-reactivity with the ZIKV E protein or with cellular proteins. Moreover, the pan-Flavivirus antibodies, with the exception of a weak signal from one of the DENV type 1 samples for Ab NR-50327, did not recognize any of the ZIKV or DENV samples. This can be partially explained by the fact that some of these antibodies (NR-4757, NR255, NR-50327) might recognize structured epitopes and their activity in denaturing PAGE/western blot assays had not been previously tested.

Overall, our data indicate that only a subset of the commercially available antibodies can be reliably utilized in the analysis of ZIKV and DENV samples given the marked cross-reactivity observed in four of the ZIKV specific antibodies and the fact that one third of the tested antibodies were unable to recognize any of the tested ZIKV or DENV strains.

### Validation of a ZIKV/DENV antibody panel

A panel of five antibodies was selected based on their specificity and affinity as determined by the western blot data presented in Figs. S5, S6 and Tables 1, 2. The antibodies selected can be divided into three categories: (i) three anti-ZIKV E protein specific antibodies (BF1176-56, GTX634157, GTX133314), (ii) one anti-DENV E serotype 2 protein specific antibodies (GTX127277) and iii) one antibody that can recognize both the ZIKV and DENV E proteins (GTX133326). Antibodies that showed a high affinity for the ZIKV E protein but also recognized other unknown antigens, possibly of cellular origin, were excluded from the panel.

**Figure 1:**
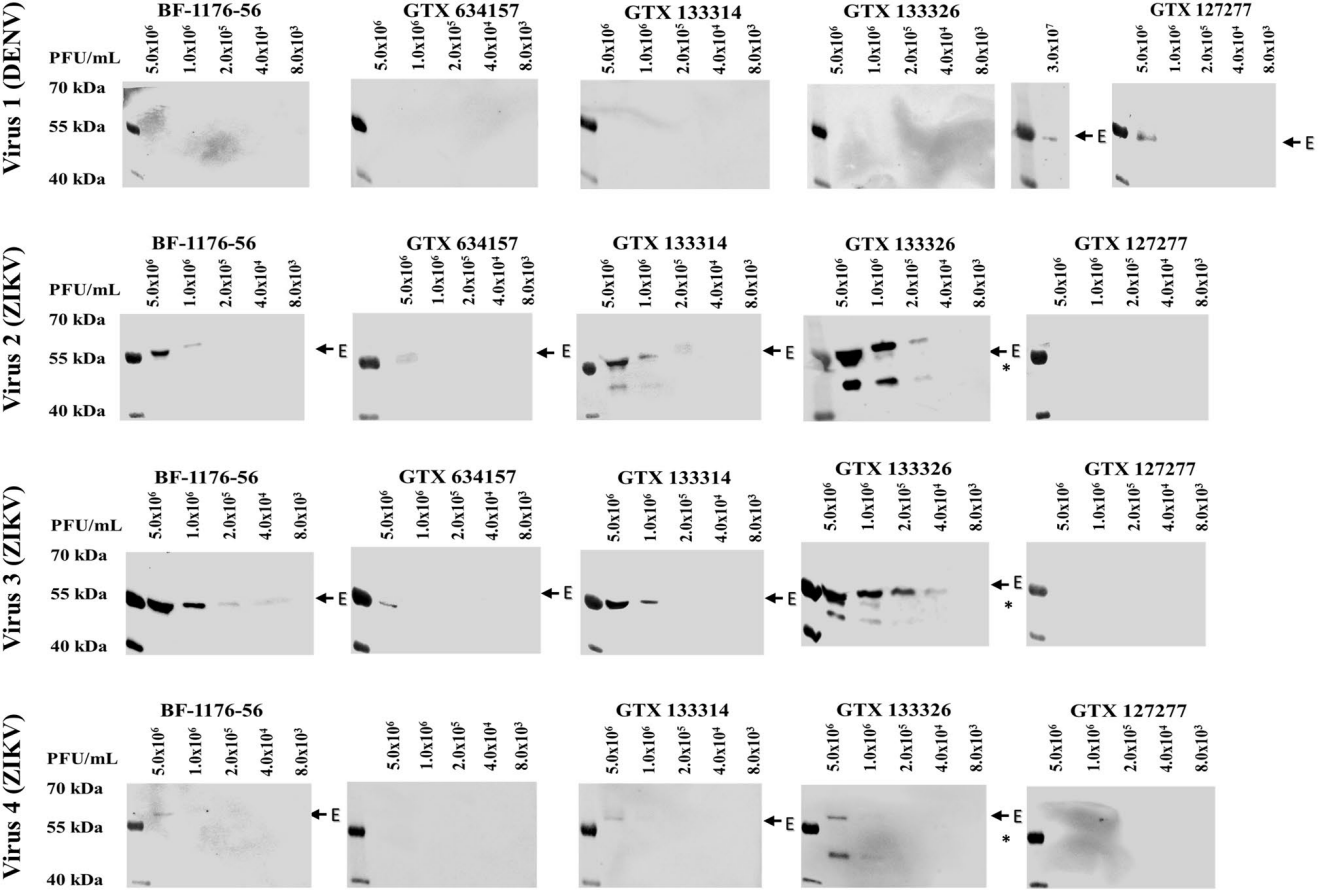
Specificity of the antibody panel for three ZIKV and one DENV viral samples. All the samples were serially diluted from 5 × 10^6^ PFU/mL to 8 × 10^3^ PFU/mL and then analyzed by SDS-PAGE/Western Blot. Arrows point to DENV/ZIKV E protein (54 kDa). *Nonspecific binding to lower MW protein.

The antibodies selected were validated utilizing three ZIKV and one DENV viral samples of known titer. (Figs. 1, S7 and data Summary in Table 3). Each viral preparation was analyzed at different concentrations (from 5 × 10^6^ to 8 × 10^3^ FFU/mL or PFU/mL) to determine both the specificity and sensitivity of the antibody panel. The testing of the viral samples carried out with the ZIKV/DENV antibody panel identified the DENV (sample 1) and three ZIKV (samples 2,3,4) viral preparations. The limit of detection for each antibody, determined by western blot, is shown in Table 4, with a lower limit varying for each antibody and virus tested and ranging from 4.0 × 10^4^ PFU/mL (for ZIKV specific and ZIKV + DENV specific antibodies) to 5.0 × 10^6^ FFU/mL for DENV specific antibodies. Overall the panel of antibody we selected was able to accurately specify and differentiate between ZIKV and DENV type 2 infected samples.

**Table 3:** Sensitivity of the anti-ZIKV/DENV antibody panel.

	Ab specificity	5.0 × 10^6^ FFU/mL	1.0 × 10^6^ FFU/mL	2.0 × 10^5^ FFU/mL	4.0 × 10^4^ FFU/mL	8.0 × 10^3^ FFU/mL
**Virus 1 (DENV)**						
BF-1176-56	ZIKV	–	–	–	–	–
GTX634157	ZIKV	–	–	–	–	–
GTX133314	ZIKV	–	–	–	–	–
GTX133326	ZIKV + DENV	– (+)^$^	–	–	–	–
GTX127277	DENV	+ +	–	–	–	–

	Ab specificity	5.0 × 10^6^ PFU/mL	1.0 × 10^6^ PFU/mL	2.0 × 10^5^ PFU/mL	4.0 × 10^4^ PFU/mL	8.0 × 10^3^ PFU/mL
**Virus 2 (ZIKV)**						
BF-1176-56	ZIKV	+ + +	+	–	–	–
GTX634157	ZIKV	+	–	–	–	–
GTX133314	ZIKV	+ + +	+	+	–	–
GTX133326*	ZIKV + DENV	+ + +	+ + +	+	–	–
GTX127277	DENV	–	–	–	–	–
**Virus 3 (ZIKV)**						
BF-1176-56	ZIKV	+ + +	+ + +	+	+	–
GTX634157	ZIKV	+	–	–	–	–
GTX133314	ZIKV	+ + +	+	–	–	–
GTX133326*	ZIKV + DENV	+ + +	+ + +	+ +	+	–
GTX127277	DENV	–	–	–	–	–
**Virus 4 (ZIKV)**						
BF-1176-56	ZIKV	+	–	–	–	–
GTX634157	ZIKV	–	–	–	–	–
GTX133314	ZIKV	+	–	–	–	–
GTX133326*	ZIKV+ DENV	+ +	+	–	–	–
GTX127277	DENV	–	–	–	–	–

Three ZIKV and one DENV viral samples were quantified and analyzed by SDS-PAGE/Western Blot. (–) no virus detected, (+) low detection, (++) good detection, (+++) excellent detection, ^$^3.0 × 10^7^ PFU/mL virus utilize, *Nonspecific binding to lower MW protein.

**Table 4:** Lower limit of detection for each antibodies of the antibody panel.

Antibody	Ab specificity	Virus 1 (DENV)	Virus 2 (ZIKV)	Virus 3 (ZIKV)	Virus 4 (ZIKV)
BF-1176-56	ZIKV	–	1.0 × 10^6^	4.0 × 10^4^	5.0 × 10^6^
GTX634157	ZIKV	–	5.0 × 10^6^	5.0 × 10^6^	–
GTX133314	ZIKV	–	2.0 × 10^5^	1.0 × 10^6^	5.0 × 10^6^
GTX133326	ZIKV + DENV	3.0 × 10^7^	2.0 × 10^5^	4.0 × 10^4^	1.0 × 10^6^
GTX127277	DENV	5.0 × 10^6^	–	–	–
		FFU/mL	PFU/mL	PFU/mL	PFU/mL

## Discussion and conclusion

In this study, we evaluated the reactivity of commercially available anti-DENV and anti-ZIKV E protein antibodies utilizing several DENV and ZIKV isolates. We observed that, although 13 out of 21 of the antibodies tested were reactive to one or more viral samples, eight failed to recognize viral antigens in any of the 21 viral samples tested. Viruses from different isolates were recognized with different affinity by the same antibody, this is likely to be due to both differences in the sequences and structure of the viral E protein and differences in the viral titer of the samples tested. Quantification by qPCR of the isolate NR-50355 and NR-50245 (1 × 10^8^ and 3 × 10^7^ genome copies/mL respectively, Fig. S8) confirmed a strict correlation between the western blot data (Tables 1 and 2) and viral titer. Furthermore, the signal detected with all ZIKV strains by four of the anti-Zika E protein antibodies (BF-1176-56, MBS5304716, GTX133325 and GTX133326) confirmed that the viral titer in all the ZIKV samples were sufficiently high to be easily detected by western blot. Nevertheless, it is plausible that other types of assays or different experimental conditions might result in the detection of the viral proteins with some of the antibodies and failed to work in our assays. Moreover, the E protein of three DENV isolates (NR-86, NR-49757 and NR-84) was not recognized by any of the DENV specific antibody tested, although, a band corresponding to a higher MW cellular protein was observed with three of the ZIKV antibodies (Fig S6). However, additional dengue antibodies can be tested in the future. Overall, we validated 3 anti-DENV E protein antibodies, 6 anti-ZIKV E protein antibodies and 4 antibodies that recognized both DENV and ZIKV E proteins. We selected a panel of 5 antibodies (3 ZIKV specific, 1 DENV specific and one cross-reactive for both ZIKV and DENV) for their specificity and sensitivity. The antibody panel was validated utilizing three ZIKV and one DENV samples. Analysis of the viral samples correctly identified the DENV and ZIKV viruses with an upper limit of detection of roughly 5.0 × 10^6^ PFU/mL.

Currently, the FDA emergency-use-authorized and CDC-developed IgM antibody capture enzyme-linked immunosorbent assay (MAC-ELISA) is considered as the gold standard for serological fluids testing for ZIKV, although a PRNT test is required to confirm the positive results^32,40^. The panel of antibodies that were reported here can also be used for developing rapid ZIKV specific, and possibly DENV specific if other DENV-specific antibodies are added to it, detection and quantification assays, to be used at port of entry, urgent care centers, and other resource-limited settings if integrated with technologies such as microfluidic channeled^41^, optical photonic crystal^42^, colorimetric analysis^43–47^, and plasmon resonance^48^.

## Supplementary information

Supplementary Information
