# Supplementary material for: An antibody panel for highly specific detection and differentiation of Zika virus
Source: Sci Rep. 2020 Jul 17;10:11906. doi: 10.1038/s41598-020-68635-6 (PMC7367842; doi:10.1038/s41598-020-68635-6)
Supplement: Supplementary file 1 — Supplementary Information [file 41598_2020_68635_MOESM1_ESM.pdf]

Supplementary Information

An antibody panel for highly specific detection and differentiation of Zika virus

Md Alamgir Kabir,[1,2] Ruben Soto-Acosta,[3] Sandhya Sharma,[1,2] Shelton S. Bradrick,[3] Mariano A. Garcia-Blanco,[3,4] Massimo Caputi, [5,*] Waseem Asghar[1,2,6] *

[1]Department of Computer & Electrical Engineering and Computer Science, Florida Atlantic University, Boca Raton, FL 33431, USA

[2]Asghar-Lab, Micro and Nanotechnology in Medicine, College of Engineering and Computer Science, Boca Raton, FL 33431, USA

[3]Department of Biochemistry & Molecular Biology, University of Texas Medical Branch, Galveston, TX, USA

[4]Duke-NUS Medical School, Singapore.

[5]Department of Biomedical Science, Charles E. Schmidt College of Medicine, Florida Atlantic University, Boca Raton, FL 33431, USA

[6]Department of Biological Sciences (Courtesy Appointment), Florida Atlantic University, Boca Raton, FL 33431, USA

* Corresponding Authors Email: mcaputi@health.fau.edu; wasghar@fau.edu

Table S1: List of antibodies tested in this study. The type, specificity, supplier, dilution, and known western blot application status, as provided by the supplier, are indicated. * ab214335 is a pan-flavivirus antibody specific for DENV type 1, 2, 3 and 4, Japanese Encephalitis virus, West Nile virus, and ZIKV. ‡ NR-50327 is a pan-flavivirus antibody specific for DENV type 2 and 3, West Nile virus, yellow fever virus, and ZIKV.

Catalog No	Description	Type	Specificity	Source	Dilutions	WB applications
**NR-2556**	Anti-Dengue E protein	Mouse monoclonal	DENV Type 2	BEI resources	1:375	Untested
**10-1706**	Anti-Dengue E protein	Mouse monoclonal	DENV Type 2	Fitzgerald	1:37.5	Untested
**NR-4757**	Anti-Dengue E protein	Mouse monoclonal	DENV Type 1	BEI resources	1:375	Untested
**10-1435**	Anti-Dengue E protein	Mouse monoclonal	DENV type 1, 2, 3 and 4	Fitzgerald	1:375	Yes
**GTX629116**	Anti-Dengue E protein	Mouse monoclonal	DENV Type 2	Genetex	1:1000	Yes
**GTX127277**	Anti-Dengue E protein	Rabbit polyclonal	DENV Type 2	Genetex	1:1000	Yes
**GTX629117**	Anti-Dengue E protein	Mouse monoclonal	DENV Type 2	Genetex	1:1000	Yes
**ab80914**	Anti-Dengue E protein	Mouse monoclonal	DENV Type 2	Abcam	1:375	Yes
**ab214335**	Anti-Flavivirus E protein	Mouse monoclonal	*	Abcam	1:37.5	Yes
**NR-50327**	Anti-Flavivirus E protein	Mouse monoclonal	‡	BEI resources	1:375	Untested
**NR-50414**	Anti-Zika E protein	Mouse monoclonal	Unknown	BEI resources	1:375	Yes

**GTX133314**	Anti-Zika E protein	Rabbit polyclonal	Unknown	Genetex	1:375	Yes
**GTX634155**	Anti-Zika E protein	Mouse monoclonal	Unknown	Genetex	1:375	Yes
**BF-1176-56**	Anti-Zika E protein	Mouse monoclonal	Unknown	Biofronttech	1:375	Yes
**MBS5304716**	Anti-Zika E protein	Mouse monoclonal	Unknown	Mybiosource	1:375	Untested
**GTX133325**	Anti-Zika E protein	Rabbit polyclonal	Unknown	Genetex	1:375	Yes
**GTX133326**	Anti-Zika E protein	Rabbit polyclonal	Unknown	Genetex	1:375	Yes
**GTX634157**	Anti-Zika E protein	Mouse monoclonal	Unknown	Genetex	1:375	Yes
**367950**	Anti-Zika E protein	Mouse monoclonal	Unknown	US Biological	1:75	Untested
**10-2715**	Anti-Zika E protein	Mouse monoclonal	Unknown	Fitzgerald	1:37.5	Untested
**10-2714**	Anti-Zika E protein	Mouse monoclonal	Unknown	Fitzgerald	1:37.5	Untested

Table S2: Viruses utilized in this study. The virus subtype, origin, validation method, viral titer, and supplier are indicated.

Cat. No	Virus	Origin / Strain	Identification	TCID (per mL)	Source
**VR-1586**	Dengue virus type 1	TH-S-man	IFA	8.9×10^{6}	ATCC
**NR-3787**	Dengue virus type 1	Costa Rica / BC89-94	IFA/ RT-PCR	8.9×10^{7}	BEI resources
**NR-3782**	Dengue virus type 1	India / 276RKI	IFA/ RT-PCR	8.9×10^{7}	BEI resources
**NR-82**	Dengue virus type 1	Hawaii	IFA/ RT-PCR	8.9×10^{4}	BEI resources
**VR-1584**	Dengue virus type 2	New Guinea C	RT-PCR	2.8×10^{4}	ATCC
**NR-12217**	Dengue virus type 2	Mexico / 328298	IFA/ RT-PCR	1.6×10^{7}	BEI resources
**NR-49750**	Dengue virus type 2	Puerto Rico / PR06-65-361	RT-PCR	2.8×10^{6}	BEI resources
**NR-84**	Dengue virus type 2	New Guinea C	IFA/RT-PCR	8.9×10^{5}	BEI resources
**NR-80**	Dengue virus type 3	Philippines/H87	IFA/RT-PCR	8.9×10^{4}	BEI resources
**NR-3798**	Dengue virus type 3	Malaysia / BC14-97	IFA/RT-PCR	8.9×10^{5}	BEI resources
**NR-86**	Dengue virus type 4	H241	IFA/RT-PCR	2.32×10^{5}	BEI resources
**NR-49757**	Dengue virus type 4	Puerto Rico / PR06-65-740	IFA/RT-PCR	2.8×10^{6}	BEI resources
**NR-50234**	Zika virus	Thailand / PLCal_ZV	N/A	1.6×10^{7}	BEI resources
**VR-1838**	Zika virus	Uganda / MR 766	RT-PCR.	8.9×10^{7}	ATCC
**VR-1843**	Zika virus	Puerto Rico / PRVABC59	RT-PCR	2.8×10^{7}	ATCC
**NR-50183**	Zika virus	Colombia/FLR	N/A	8.9×10^{7}	BEI resources
**NR-50245**	Zika virus	Malaysia / P6-740	N/A	2.8×10^{7}	BEI resources
**NR-50280**	Zika virus	Mexico / MEX 2-81	IFA	8.9×10^{6}	BEI resources
**NR-50355**	Zika virus	Honduras / R103451	N/A	1.6×10^{8}	BEI resources

**NR-50066**	Zika virus	Nigeria / IbH 30656	N/A	1.6×10^7	BEI resources
**NR-50551**	Zika virus	Mexico / R116265	N/A	2.8×10^7	BEI resources

IFA - Indirect Fluorescent Antibody Assay, RT-PCR- Reverse transcriptase polymerase chain reaction, N/A- Not Available , and TCID50 (Median Tissue Culture Infectious Dose)

a)

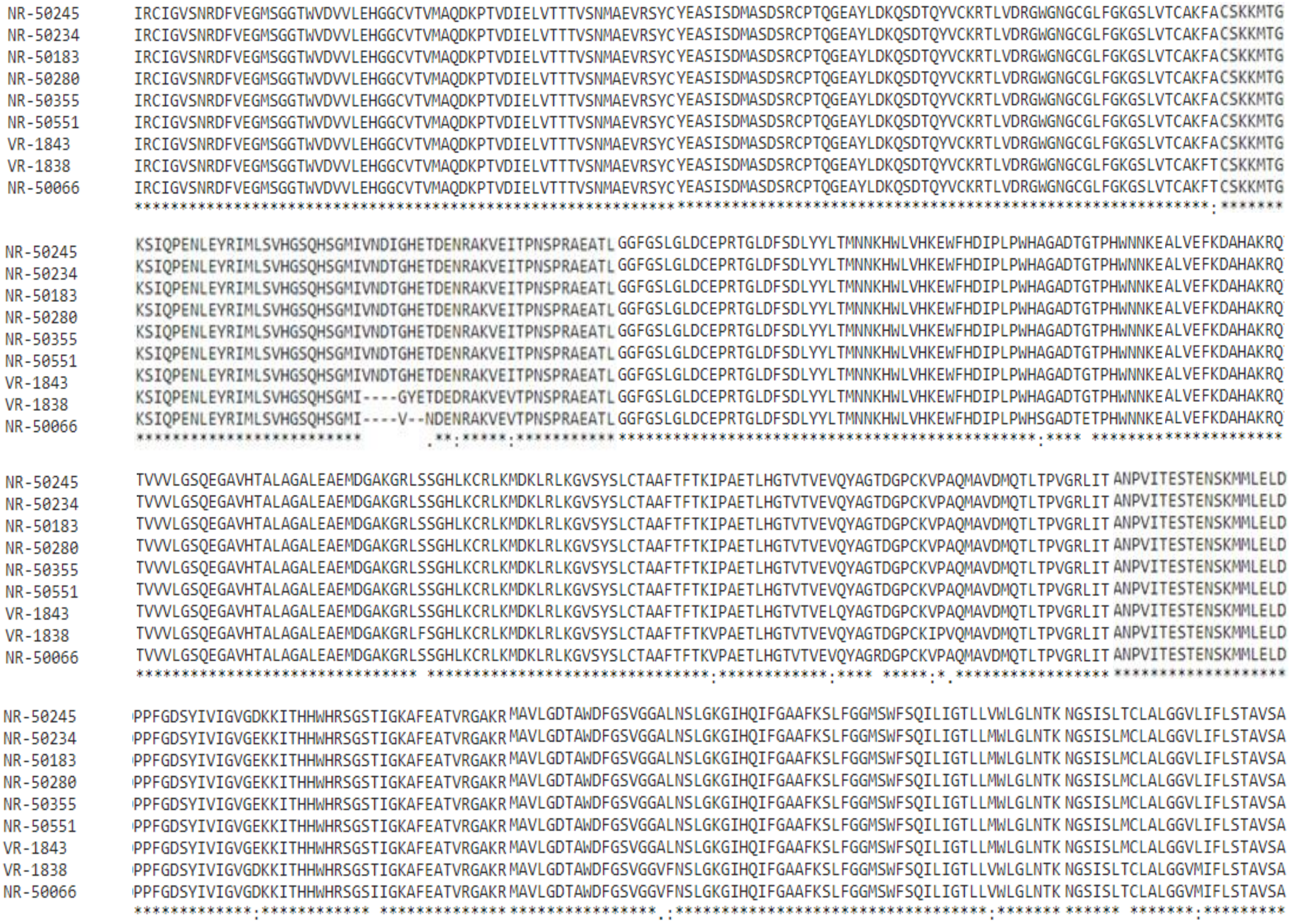

b)

	NR-50245	NR-50234	NR-50183	NR-50280	NR-50355	NR-50551	VR-1843	VR-1838	NR-50066
1: NR-50245	100.00	99.21	99.21	99.21	99.21	99.21	99.01	97.80	97.59
2: NR-50234	99.21	100.00	100.00	100.00	100.00	100.00	99.80	97.20	96.99
3: NR-50183	99.21	100.00	100.00	100.00	100.00	100.00	99.80	97.20	96.99
4: NR-50280	99.21	100.00	100.00	100.00	100.00	100.00	99.80	97.20	96.99
5: NR-50355	99.21	100.00	100.00	100.00	100.00	100.00	99.80	97.20	96.99
6: NR-50551	99.21	100.00	100.00	100.00	100.00	100.00	99.80	97.20	96.99
7: VR-1843	99.01	99.80	99.80	99.80	99.80	99.80	100.00	97.00	96.79
8: VR-1838	97.80	97.20	97.20	97.20	97.20	97.20	97.00	100.00	97.99
9: NR-50066	97.59	96.99	96.99	96.99	96.99	96.99	96.79	97.99	100.00

Figure S1: Analysis of amino acid sequences for all ZIKV strains used. a) Multiple alignment of 504 amino acid sequences of each ZIKV E protein. (*) represents the same amino acid sequence.(:) represents the different amino acid sequences with similar properties (.) represents the different amino acid sequences with weakly similar properties. b) Percent Identity Matrix based on 504 amino acids created by Clustal 2.1 for all ZIKV strains.

a)

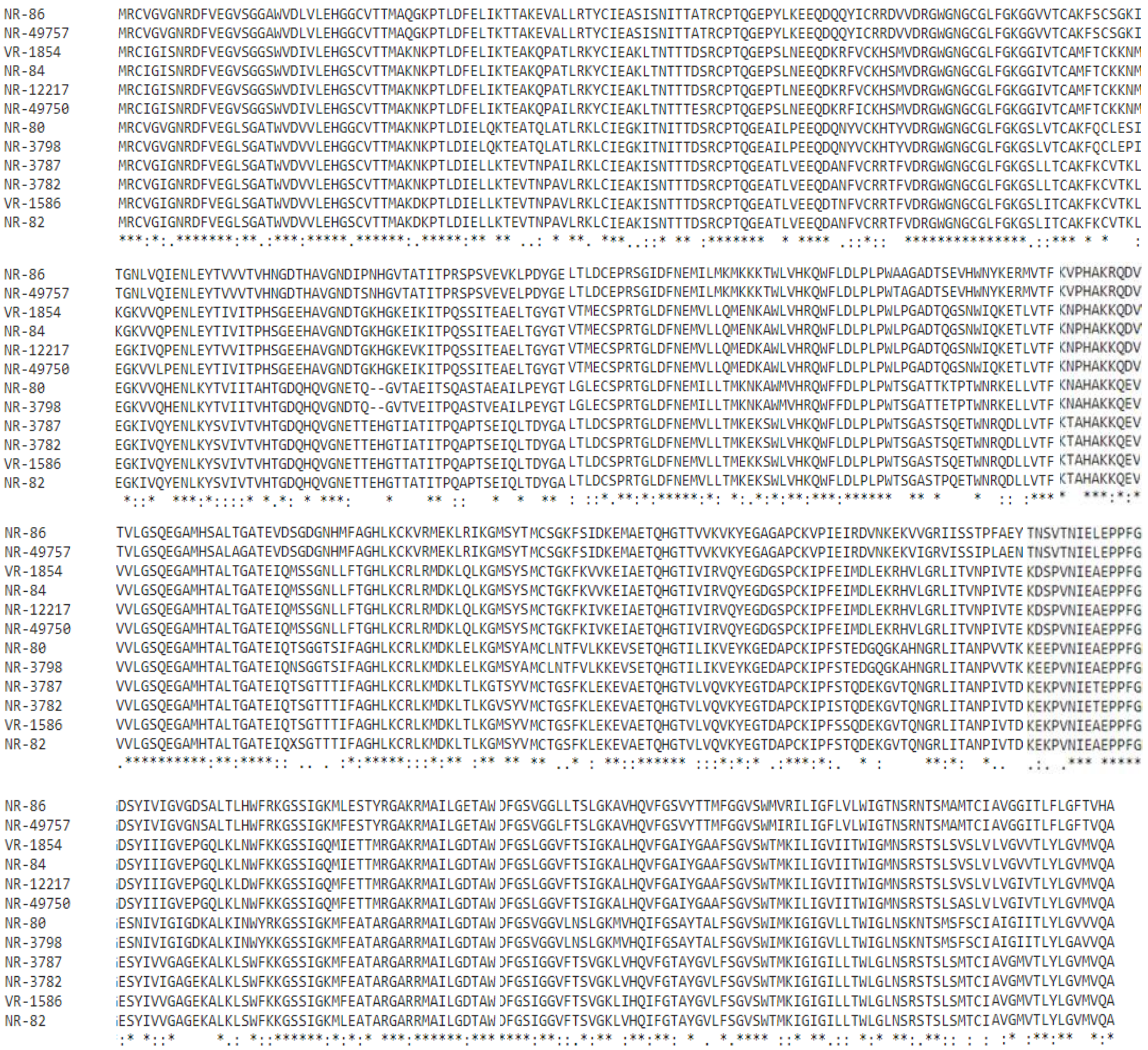

b)

	NR-86	NR-49757	VR-1854	NR-84	NR-12217	NR-49750	NR-80	NR-3798	NR-3787	NR-3782	VR-1586	NR-82
1: NR-86	100.00	96.77	63.43	63.43	63.84	63.64	62.88	63.29	63.03	63.43	63.03	63.23
2: NR-49757	96.77	100.00	63.84	63.84	64.44	64.24	63.08	63.49	63.84	64.24	63.84	63.64
3: VR-1854	63.43	63.84	100.00	100.00	97.98	97.98	68.15	68.56	68.48	68.48	68.69	68.48
4: NR-84	63.43	63.84	100.00	100.00	97.98	97.98	68.15	68.56	68.48	68.48	68.69	68.48
5: NR-12217	63.84	64.44	97.98	97.98	100.00	97.98	68.36	68.97	69.49	69.49	69.70	69.29
6: NR-49750	63.64	64.24	97.98	97.98	97.98	100.00	67.75	68.15	68.48	68.28	68.48	68.08
7: NR-80	62.88	63.08	68.15	68.15	68.36	67.75	100.00	97.97	77.48	77.48	77.48	77.69
8: NR-3798	63.29	63.49	68.56	68.56	68.97	68.15	97.97	100.00	77.28	77.28	77.28	77.69
9: NR-3787	63.03	63.84	68.48	68.48	69.49	68.48	77.48	77.28	100.00	99.19	97.58	97.98
10: NR-3782	63.43	64.24	68.48	68.48	69.49	68.28	77.48	77.28	99.19	100.00	97.37	97.78
11: VR-1586	63.03	63.84	68.69	68.69	69.70	68.48	77.48	77.28	97.58	97.37	100.00	98.38
12: NR-82	63.23	63.64	68.48	68.48	69.29	68.08	77.69	77.69	97.98	97.78	98.38	100.00

Figure S2: Analysis of amino acid sequences for all DENV strains used. a) Multiple alignment of 495 amino acid sequences of each DENV E protein. (*) represents the same amino acid sequence.(:) represents the different amino acid sequences with similar properties (.) represents

the different amino acid sequences with weakly similar properties. b) Percent Identity Matrix based on 495 amino acids created by Clustal 2.1 for all DENV strains.

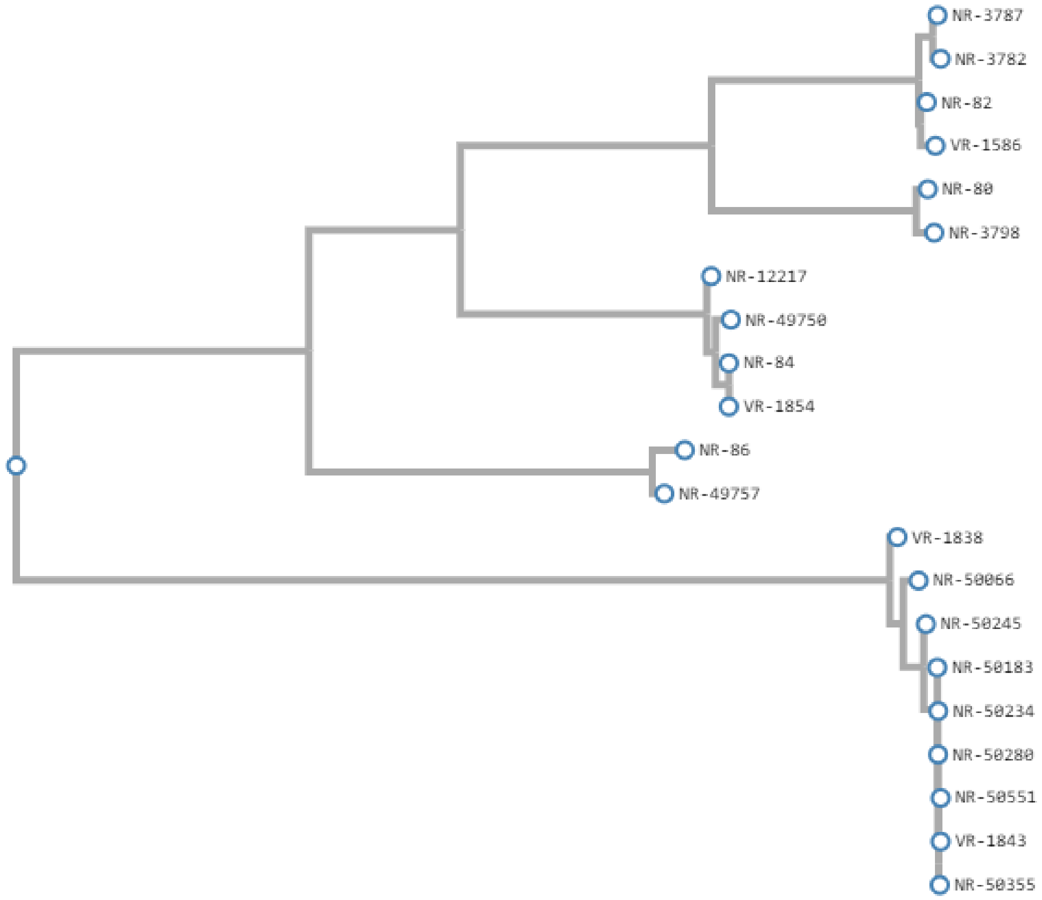

Figure S3: Phylogenetic tree of the ZIKV and DENV isolates considering the amino acid sequences of each ZIKV/DENV E protein used in this study.

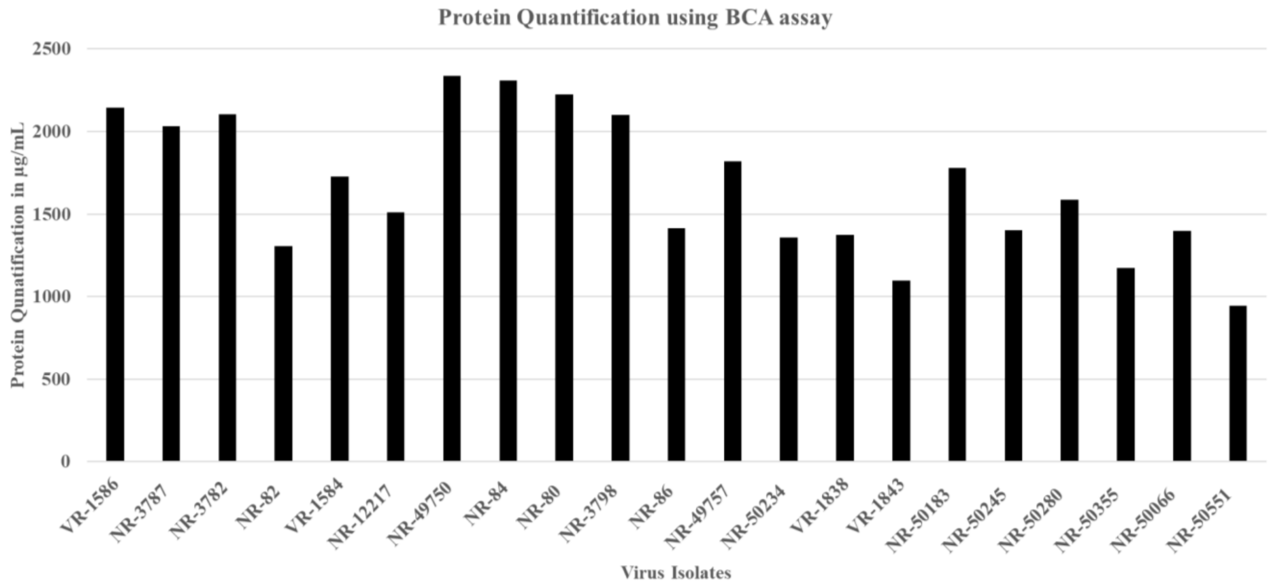

Figure S4: BCA protein quantification assay of the viral lysate. 15µL of viral sample was used for quantifying the amount to total protein content in each viral lysate.

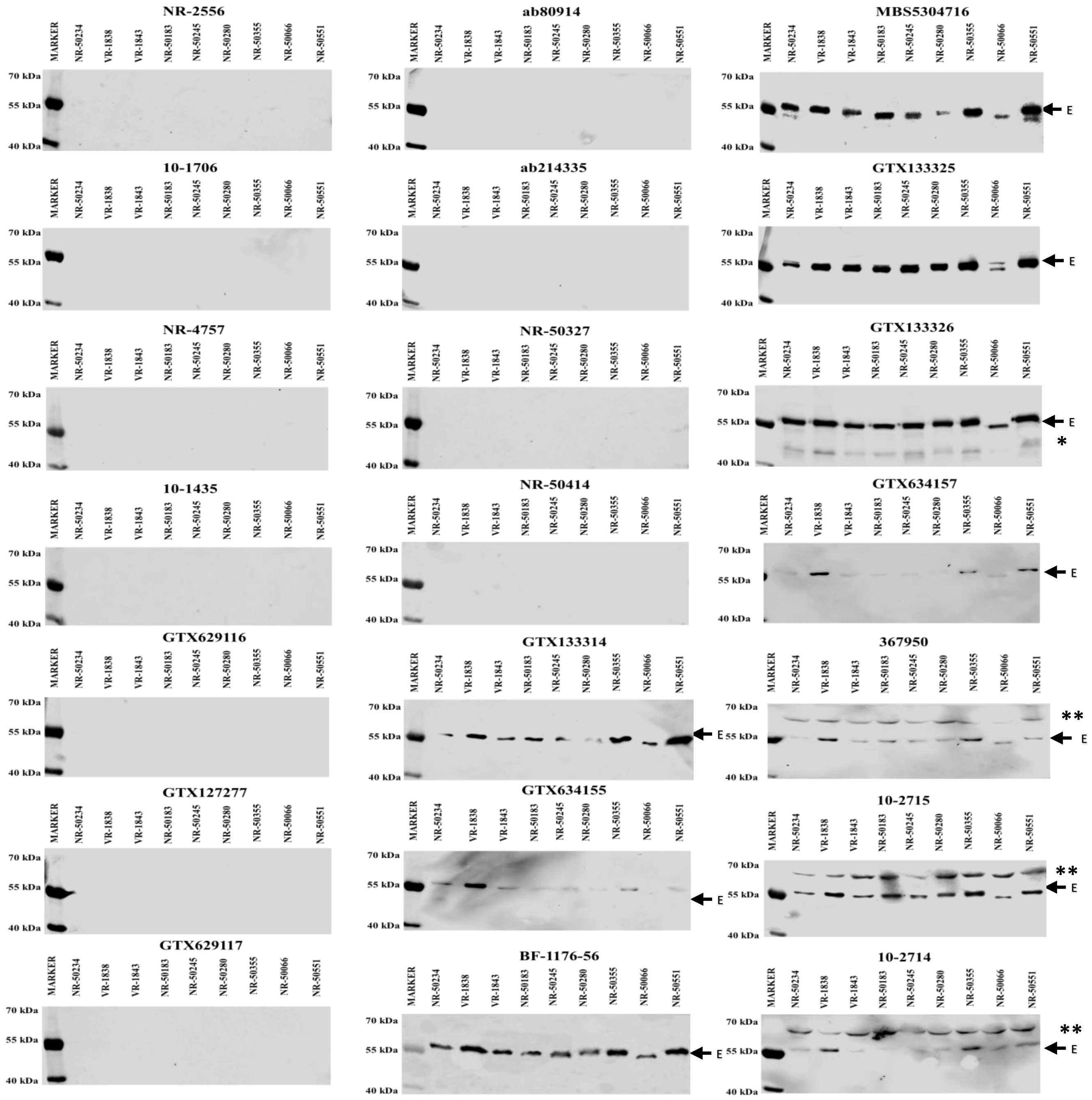

Figure S5: Specificity and cross-reactivity of 21 antibodies against 9 ZIKV strains is probed by SDS-PAGE/Western Blot. Arrows indicate the ZIKV E protein at approximately 54 kDa. (**) Nonspecific binding to higher MW cellular protein; (*) Nonspecific binding to lower MW protein.

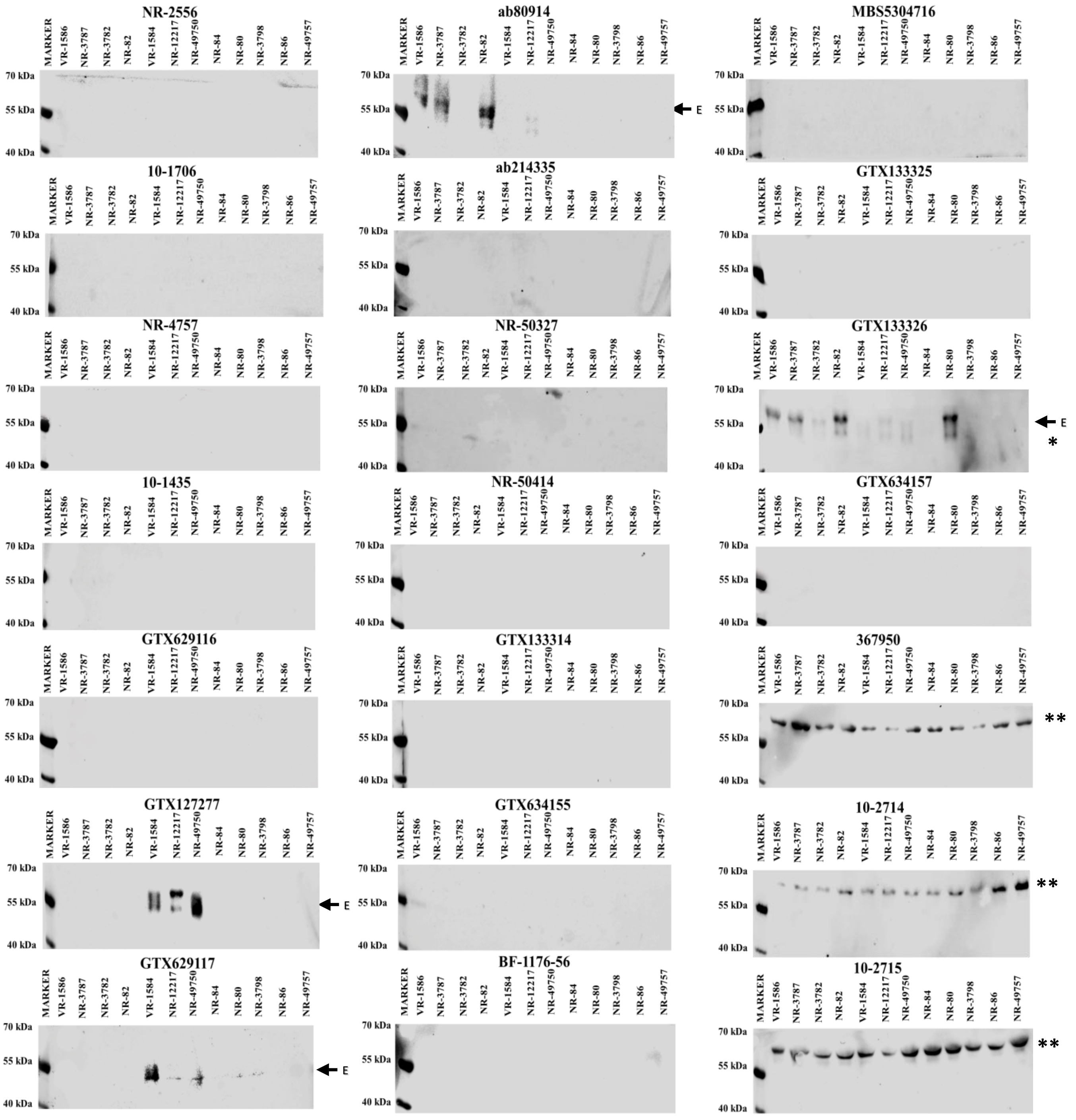

Figure S6: Specificity and cross-reactivity of 21 antibodies against 12 DENV strains is probed by SDS-PAGE/Western Blot. Arrows indicate DENV E protein at approximately 54 kDa. (**) Nonspecific binding to higher MW cellular protein; (*) Nonspecific binding to lower MW protein.

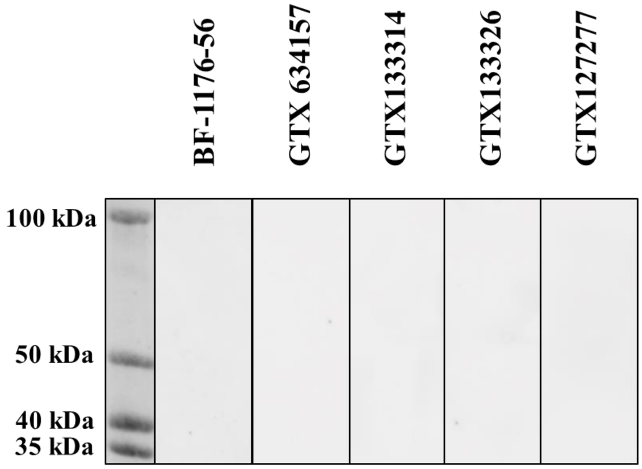

Figure S7: Non-reactivity of selected paneled antibodies against virus-free Vero cells supernatant determined by SDS-PAGE/Western Blot.

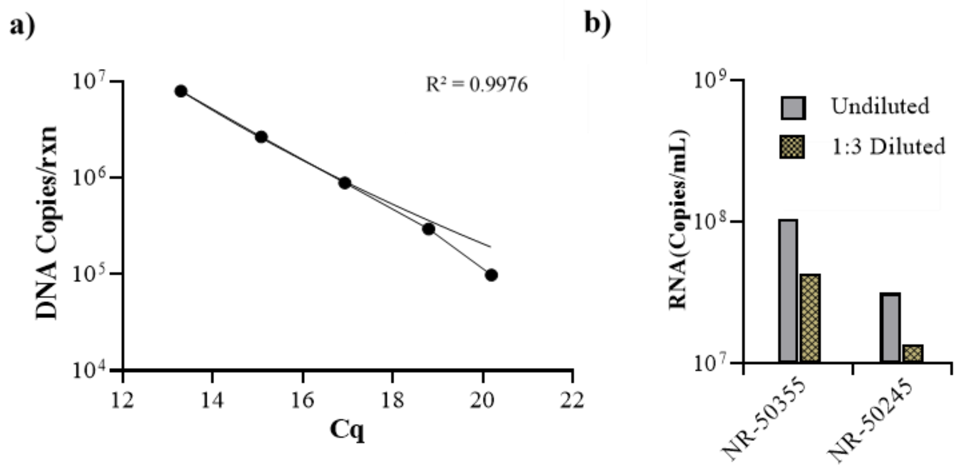

Figure S8: RNA was isolated from two ZIKV isolates and quantified using RT-qPCR (see methods). a) Copy numbers of serially diluted synthetic DNA standards were plotted against the corresponding quantification cycle (Cq) values to produce standard quantification curve. b) Viral RNA copies are calculated using the standard curve.
